# DLS: A Link Prediction Method Based on Network Local Structure for Predicting Drug-Protein Interactions

**DOI:** 10.3389/fbioe.2020.00330

**Published:** 2020-04-24

**Authors:** Wei Wang, Hehe Lv, Yuan Zhao, Dong Liu, Yongqing Wang, Yu Zhang

**Affiliations:** ^1^Department of Computer Science and Technology, College of Computer and Information Engineering, Henan Normal University, Xinxiang, China; ^2^Big Data Engineering Laboratory for Teaching Resources and Assessment of Education Quality, Xinxiang, China

**Keywords:** drug-protein interactions, network analysis, link prediction, DPI local structural similarity, network local structure

## Abstract

The studies on drug-protein interactions (DPIs) had significant for drug repositioning, drug discovery, and clinical medicine. The biochemical experimentation (*in vitro*) requires a long time and high cost to be confirmed because it is difficult to estimate. Therefore, a feasible solution is to predict DPIs efficiently with computers. We propose a link prediction method based on drug-protein interaction (DPI) local structural similarity (DLS) for predicting the DPIs. The DLS method combines link prediction and binary network structure to predict DPIs. The ten-fold cross-validation method was applied in the experiment. After comparing the predictive capability of DLS with the improved similarity-based network prediction method, the results of DLS on the test set are significantly better. Moreover, several candidate proteins were predicted for three approved drugs, namely captopril, desferrioxamine and losartan, and these predictions are further validated by the literature. In addition, the combination of the Common Neighborhood (CN) method and the DLS method provides a new idea for the integrated application of the link prediction method.

## Introduction

The drug-protein interaction (DPI) prediction plays an indispensable role in discovering new functions of drugs. The traditional drug development is time-consuming, labor intensive, and low in success rate. *In silico* prediction of DPIs can accelerate drug research and development without increasing the risk of failure (Ashburn and Thor, 2004). DPI predictions can reveal possible interactions between drugs and proteins, and identify potential new functions for drugs. For example, a drug sildenafil was originally intended to treat cardiovascular disease. Drug-target interaction predictions have found that sildenafil can stimulate penile erections. Therefore, the new function of sildenafil is to treat male erectile dysfunction (Boolell et al., 1996). Another successful case is thalidomide, which was developed to treat sedation but later used in the treatment of diabetes (Amirshahrokhi and Ghazi-Khansari, 2012).

At present, these methods for predicting DPIs are mainly based on drug similarity and protein similarity (Zhang et al., 2018). These methods require characteristic information of drugs, proteins, and DPI, such as chemical structure, genomic sequence, type of binding, reason for interaction, etc. When the above characteristic information is not available, these methods cannot be effectively executed. For example, Keizer used chemical two-dimensional (2D) structural similarity to predict new targets for known drugs and confirmed that five of the 23 new drug target associations were valid (Keiser et al., 2009). Methods based on protein sequence similarity have also been applied in drug-protein interaction prediction (Bleakley and Yamanishi, 2009), such as using protein sequence similarity as the basis of classification rules for bipartite local models. At the same time, DTI predictions based on similarities between protein sequences or drug structures have limitations since its underlying assumption that similar drugs share similar targets is not necessarily true (Ding et al., 2014).

The DPI can be expressed in the form of bipartite network, with drugs and proteins forming two disjoint sets of nodes and the interactions between the drugs and proteins forming the edges (Chen et al., 2018; Wu et al., 2018; Ma et al., 2019). At present, the bipartite network has made significant achievements in the research of drug repositioning, drug-disease association analysis, drug-protein interaction prediction, and gene-disease association prediction (Wang et al., 2014; Sun, 2015; Zhang et al., 2017, 2018, 2019; Yue et al., 2019). Lee proposed a method for drug repositioning using integrated networks to achieve excellent performance (Lee and Yoon, 2018). Zhang proposed an inference method based on network topology similarity to predict unobserved drug-disease associations (Zhang et al., 2018). Cheng proposed a network-based inference (NBI) method that used only the binary similarity of the target’s topological network to infer novel proteins for known drugs (Cheng et al., 2012). Zhang proposed a network link inference method based on linear neighborhood similarity to predict miRNA-disease associations (Zhang et al., 2019b). These network analysis methods provide ideas for DPI network research.

Link prediction is a crucial content of network analysis that has received widespread attention (Almansoori et al., 2012). The potentiality of establishing links between two nodes that have not yet been attached is predicted by known network nodes and structure information. The current link prediction method is widely used in DPI prediction and drug repositioning because it only requires topology information in the network. In terms of drug side effects, a drug side-effect prediction framework based on link prediction has been established (Luo et al., 2014). At the same time, the application of link prediction method in heterogeneous networks overcomes the problem of high feature dimension in traditional machine learning (Stanfield et al., 2017). In addition, drug sensitivity has been represented as a link prediction problem. For example, Turki applies link prediction to cancer drug sensitivity prediction, and the proposed two link prediction algorithms are more predictive and stable than current prediction algorithms (Turki and Wei, 2017). At the same time, integrated applications of prediction methods have also been to predict ligand-target interactions (Gong et al., 2019; Zhang et al., 2019).

The similarity-based method is considered to be the simplest link prediction framework, which measures a score for each pair of unlinked nodes, which is defined as the similarity between the nodes (Wang et al., 2013). All unobserved links are ranked according to their scores and the higher the score, the higher the likelihood of similarity. At present, similarity-based methods are widely used in biological network research. Chen develops a similarity-based approach to predict the target group of drug, and providing a series of candidate targets for each drug (Chen and Zeng, 2013). Dai proposes a link prediction algorithm based on relational similarity, which can obtain higher quality prediction results than other similar algorithms (Dai et al., 2017). Zong proposes a similarity-based method for drug-target prediction, which provides a promising solution for drug target prediction in heterogeneous networks (Zong et al., 2017).

In this paper, we propose a novel prediction method named DLS, which is based on local topologies in the DPI network and can more effectively predict unobserved DPIs. Firstly, the DPI network is constructed that is based on known DPIs. Secondly, the local topology of the network is analyzed. Thirdly, an effective prediction of DPIs is achieved based on link prediction methods. We compared the performance of this method with the formed baseline methods (CN, JA, and PA) on six metrics (AUC, AUPR, precision, sensitivity, F1-score, accuracy). The results show that the DLS method performs better than the baseline method on six indicators. Furthermore, a comprehensive prediction of DPIs was made using our method, and the reliability of some results was verified by the literature.

## Materials and Methods

### Materials

The MATADOR database^1^ is a free online database of DPIs, which includes interaction patterns between chemicals and proteins (Gunther et al., 2008). As of December 2019, the MATADOR database contains a total of 801 drugs and 2901 proteins. The number of possible interactions in the dataset is 2,323,701 (801×2901), and the given number of interactions is 15,843. The positive samples account for only 0.682% of all interactions. The proportion of positive samples is very low, and the drug-protein pair with actual interaction is less likely to be selected as a negative sample. In the experiment, all known drug-protein interaction pairs were considered to be positive samples. We randomly selected the same number of positive samples from the remaining non-interacting drug-protein pairs as the negative samples to avoid bias caused by imbalance problems. Here, we also analyzed the ratio of protein and drug by selected negative samples, such as Supplementary Table 1. The results show that about 86% of the drugs and 84% of proteins are covered in each random selection of sample, and the samples can basically cover the types of drugs and proteins in the data.

### Method Overview

**FIGURE 1:**
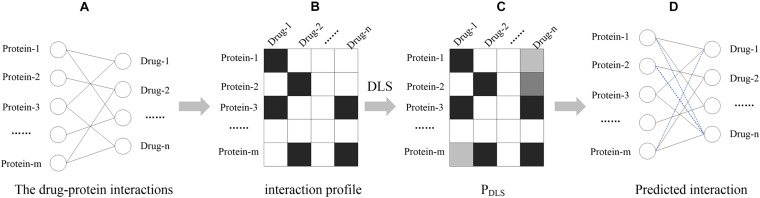
The workflow of DLS. **(A)** The drug-protein interaction network, where nodes represent proteins and drugs, and black lines represent known interactions. **(B)** Interaction profile. The relationship between drugs and proteins is represented by a matrix, and the rows and columns represent proteins and drugs, respectively. When there is an interaction between the drug and the protein, the corresponding position of the matrix *X* (*i*, *j*) = 1, which is represented by a black square box. **(C)** The similarity score calculated by DLS, where the higher the score, the darker the corresponding box color. **(D)** Predicted interaction. The blue dotted line represents the predicted potential interaction.

First of all, protein, drug and their interactions are formulated as a bipartite network, in which the vertices can be divided into two disjoint and independent sets: *U* = {*p*_1_, *p*_2_,… *p*_*m*_} and *V* = {*d*_1_, *d*_2_,… *d*_*n*_}. When the protein *p*_*i*_ in *U* has an interaction with the drug *d*_*j*_ in *V*, an edge is drawn between *p*_*i*_ and *d*_*j*_. The *m* × *n* binary matrix *X* can represent the bipartite network in which each column is a drug and each row is a protein. If the *p*_*i*_ has an interaction with the *d*_*j*_, *X*(*i*, *j*) = 1; otherwise *X*(*i*, *j*) = 0. The process is shown in Figure 1, broadly divided into the following sections. In the first step, the DPIs are expressed as binary matrix *X*, named “interaction profile.” Then, based on the drug-protein interaction profile, DLS can calculate a matrix P_*DLS*_ of potential interactions between drug-protein. Finally, the drug-protein interaction is predicted based on the output score of the DLS. The higher the score calculated by DLS, the higher the reliability of the drug has interaction with protein.

### Similarity-Based Method

We compare our method with recent work (Lu et al., 2017), which improves the similarity method, extends it to binary DPI networks, and demonstrates good performance on the MATADOR database. As mentioned earlier, the simplest framework for link prediction methods is based on similarity algorithm. The study on similarity is the mainstream problem (Lü and Zhou, 2011). The similarity-based method can be divided into node-based similarity and structural-based similarity. Since the properties of nodes are usually hidden, we focus on structural similarity, which is based entirely on the network structure. The network structure-based similarity method was originally used to calculate the similarity of nodes in a single node network. For DPI binary networks, the similarity of nodes cannot be directly calculated using the original similarity method. Next, we introduce the improved structure-based similarity measure.

The Common Neighborhood (CN) method defines the number of co-neighbors of drug-protein pairs as a drug-protein similarity. If two nodes share many common neighbors, there may be a link between the two nodes. The more neighbors of drugs and proteins, the greater the possibility of drug-protein interaction. The essence of the CN method is to calculate the total number of paths of length 2. However, in the DPI binary network, the neighbors of proteins are drugs, and the neighbors of drugs are proteins, so it is impossible for drugs and proteins to connect through a path of length 2. In the DPI network, the minimum path length for the drug-protein connection is 3. In this paper, the drug-protein connection with pathway 3 was investigated as a potential interaction.

The Jaccard (JA) method is a similarity measure commonly used in recommendation systems. It measures the probability of common features of nodes. This method considers the influence of nodes in the network and is basically a normalized version of CN. For example, influential people can naturally establish good connections with other people in a social network. Therefore, even if two influential people are not close friends, they may share many common neighbors. In this situation, the CN method will get a high score. The JA method solves this problem by placing more emphasis on the links of unaffected nodes to ensure that the common neighbors they share are due to their similarity rather than their influence (Leydesdorff, 2008).

The Preferential Attachment (PA) method defines that the probability of connecting edges between any two pairs of nodes in the network is proportional to the product of the degrees of these two nodes. The mechanism of this method can be used to generate an evolved scale-free network, where the probability of a new link connecting to node is proportional to the degree of that node. A similar mechanism may also lead to a scale-free network that does not grow, where at each time step, the old links are deleted and new links are generated. The PA method has been widely used to quantify the functional importance of links affected by various network-based dynamics (Holme et al., 2002; Yin et al., 2006; Zhang et al., 2007). The method does not require the neighborhood information of each node, so it has the smallest computational complexity.

### Network Prediction Method Based on DPI Local Structure (DLS)

**FIGURE 2:**
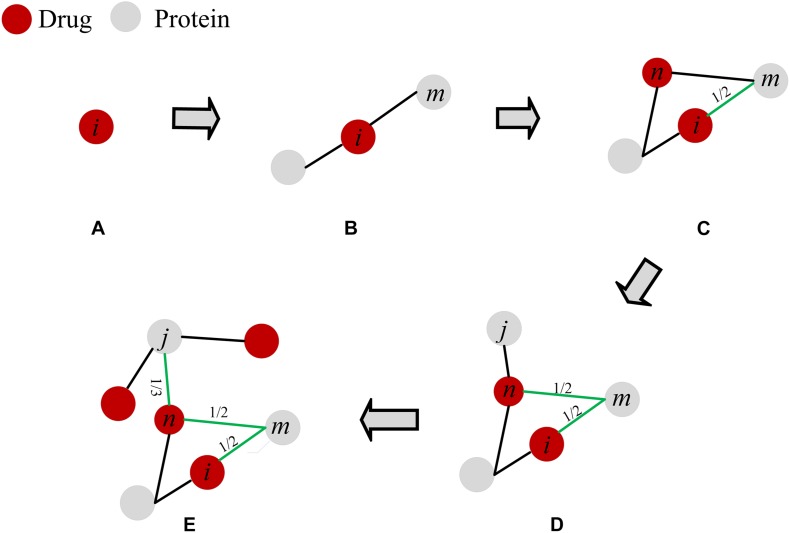
The scoring process for drug-protein interactions. **(A)** Drug. **(B)** The protein node of drug action. **(C)** The resources of drug *i* are averagely distributed to its neighboring protein nodes, where protein *m* gets 1/2 of the resources. **(D)** The protein node that received the resource is used as a base point again, and the resource of this node is evenly redistributed to the neighboring drug nodes, where *n* gets 1/2 resource of *m*. **(E)** This process is repeated until the target drug finds a neighboring protein node that is not directly connected. Finally, the scores of the branches on the path are added to obtain the predicted score. The scores of drug *i* and protein *j* is *S_*ij*_* = 1/2 + 1/2 + 1/3 = 4/3.

The above prediction methods only consider the number of common neighbor nodes or the degree of nodes in the similarity calculation, and do not consider the local structure information. In this paper, we applied a mass diffusion-based method in drug-protein interaction networks to obtain prediction scores. Each drug node averagely distributes its resource to all neighboring protein nodes and then redistributes the proteins that receive the drug resources to all neighboring drug nodes. We detail the process of this method in Figure 2. In this paper, the local structure information in the DPI network is mainly the path of drug-protein connectivity and the degree distribution. Degree and path are the most intuitive parameters in the network, and they play an important role in DPI network analysis, and they have important effects on network structure and network stability (Friedel and Zimmer, 2007). In addition, degrees and paths are the basis of many other parameters in the network. Therefore, we choose these two basic parameters as the theoretical basis. There are differences in local structural information in the network, and such local differences may affect the interaction of drugs and proteins. The method of NBI has demonstrated that local structure affects the prediction of drug-target interactions (Zhou et al., 2009, 2010; Cheng et al., 2012). Therefore, the similarity prediction method based on the local network structure should be developed. It is helpful to discover the influence of local information on drug-protein interaction in DPI networks.

Based on the influence of local structure on prediction in DPI network, we propose a network prediction method based on DPI local structure, called DPI local structure method (DLS). In the binary network, the degree of a node indicates the number of other nodes connected to the node. The degree of the drug indicates the number of proteins bound to the drug, and the degree of the protein indicates the number of drugs recognized by the protein. In the DPI network, the degree of a node can measure the difference in network structure. When other additional information is unknown, the degree is directly obtained, so we define the DLS according to the degree of the node. DLS is defined by the degree of drug and protein in the DPI local network structure, and the score of the drug (*i*) -protein (*j*) pair directly connected in the DPI network is defined as:

(1) $$D\,L\,S\,\left(i,j\right)=\frac{1}{\min\left\{K_{i},K_{j}\right\}}$$

where *K* represents the degree of the node and “1” represents the interaction between the drug and the protein. The score of the drug (*i*)-protein *(j*) pair that is not connected in the DPI network is defined as:

(2) $$S_{i\,j}=\sum_{\begin{array}{c} x\in \Gamma \,\left(i\right)\cap \Gamma^{\prime }\,\left(j\right)\\ y\in \Gamma \,\left(j\right)\cap \Gamma \,\left(x\right) \end{array}}\left(D\,L\,S\,\left(i,x\right)+D\,L\,S\,\left(x,y\right)+D\,L\,S\,\left(y,j\right)\right)$$

where Γ′(*j*) is defined as the set of neighbors of protein *j’s* neighbors, Γ(*i*) denote the set of neighbors of *i*, Γ(*j*) denote the set of neighbors of *j*.

The DLS method is based on the DP bipartite network topology and mass diffusion to predict unknown DPI. The process of mass diffusion is the diffusion of drug resources to neighboring non-interacting proteins (diffusion process: drug-protein-drug-protein). First, the resources of a given drug are evenly distributed to its neighboring protein nodes. Then, the protein node that received the resource of the previous node is used as the base point again, and the resource of this node is averagely redistributed to the neighboring drug nodes. This process is repeated until the target drug finds a neighboring protein node that is not directly connected. Finally, all the scores of the process are added to obtain the total predicted score. In our method, predictive scores are calculated for each given drug and unlinked protein, and the drug-protein interaction is determined based on the high score.

### Evaluation

The 10-fold cross-validation was used for this experimental evaluation. The performance measures used in this paper are the overall prediction Accuracy, F1-score, Precision, Sensitivity, and the AUC. The F-score can be interpreted as a weighted harmonic average of the precision and recall. The ROC curve is probably the most robust technique for evaluating classifiers and visualizing their performance. The area under the curve (AUC) is used to measure the quality of the predicted DPI. In our experiments, prediction methods were applied to the training data, and the predicted links were sorted according to their scores. As shown in Supplementary Figure 1, the experiments have determined that the DLS method has the best performance when the threshold is set to 10,000. We calculate the value of the test indicator based on the top 10,000 links predicted and then average them as the final evaluation result.

## Results

### Investigation on the Interaction Data

**FIGURE 3:**
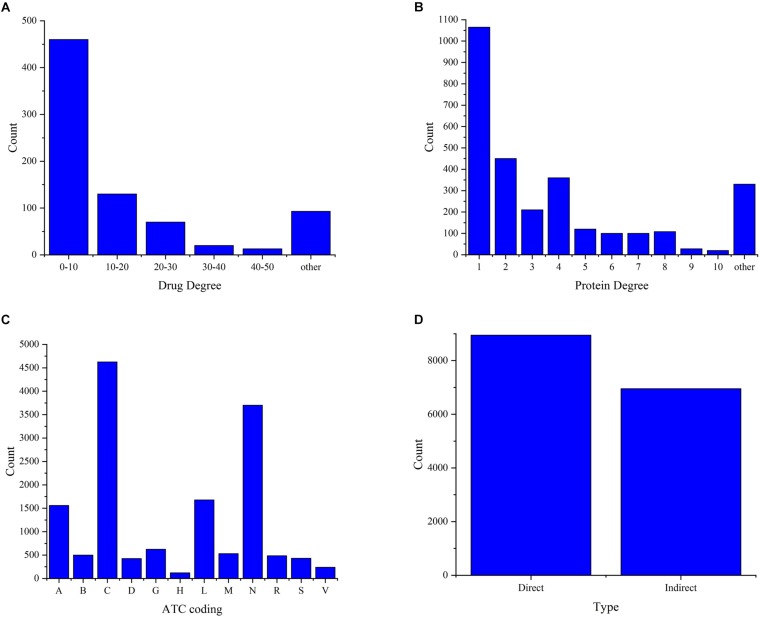
Investigation of the data set. **(A)** The degree distributions of drugs. **(B)** The degree distribution of the proteins. **(C)** The functional distributions of drugs. **(D)** The type distribution of protein-drug interactions.

The analysis of the degree distribution of drugs and proteins in the DPI network can reveal hidden information. Therefore, we constructed a drug-protein interaction network using a bipartite graph to check the degree distributions of both binding drugs and proteins (Figures 3A,B). From Figure 3A, we can see that more than 57% of the drugs bind less than ten proteins, which is consistent with the fact that the drug can bind multiple proteins but not all proteins. From Figure 3B, we can see that most of proteins bind with only one drug, indicating that the binding of drug and protein is specific. At the same time, it can be seen that a protein can have multiple drug ligands, which may be related to different diseases caused by mutation of one protein. There are 801 drugs and 2901 proteins forming 15,843 DPIs. In all, we can infer that the connections of the drug-protein bipartite graph are sparse, and the average degree of drugs is larger than that of proteins.

Figure 3C shows the functional distribution of drugs in the dataset, where more than 50% of the drugs act on the nervous system and cardiovascular system. This suggests that the dataset provides data services for the study of the nervous system and the cardiovascular system. At the same time, the proteins in this dataset may be mainly derived from these two systems. This provides some guidance for the study of pathogenesis and drug treatment of neurological diseases and cardiovascular diseases. Figure 3D shows the distribution of direct and indirect interactions of proteins and drugs in the data set, where more than 56.4% of the types are direct interactions. Although 43.6% of the types are indirect interactions, these interactions were confirmed during the text mining and manual management process. Therefore, all types of interactions are used as the experimental data.

### Performance Comparison

**FIGURE 4:**
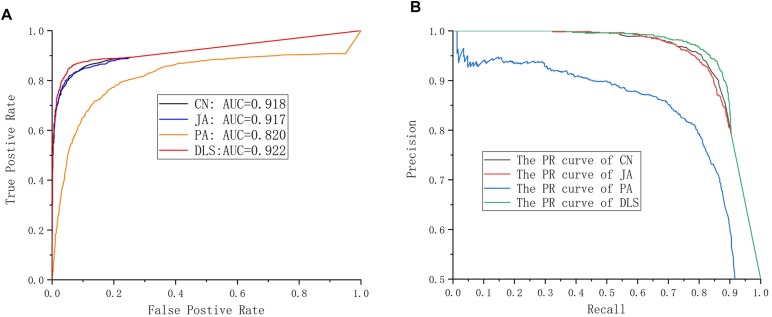
Performance evaluation. **(A)** The ROC curves for different methods. **(B)** The PR curves for different methods.

In this section, the prediction capabilities of DLS are compared with CN, JA, and PA. The results are shown in Figure 4A and Table 1. In Figure 4A, the ROC curves obtained by various methods are shown. The AUC value gained by the DLS method was 0.922, which was obviously higher than the value of AUC gained by using the CN (0.918), JA (0.917), and PA (0.820) methods, respectively. Figure 4B shows the PR curves for different methods. The AUPR value of the DLS method is 0.954, which is significantly higher than the AUPR values obtained by the CN (0.949), JA (0.948), and PA (0.844) methods. The above analysis shows that the DLS method has better prediction capabilities than the CN, PA, and JA methods. In order to confirm the reliability of the DLS method, we compared the precision, sensitivity, F1-score and accuracy of CN, JA, PA, and the DLS method, respectively. As shown in Table 1, we report the average performance of ten times running. The DLS method displays a higher performance in terms of precision, sensitivity, F1-score and accuracy, compared with CN, JA, and PA methods. At the same time, the DLS method has the smallest standard deviation, and the method is more stable. Therefore, our method is superior to other methods in situations where only information on drug and protein interactions.

**TABLE 1:** The performance of various prediction methods.

Method	Precision		Sensitivity		F1-score		Accuracy	
	Mean	Std	Mean	Std	Mean	Std	Mean	Std
CN	0.857	0.043	0.805	0.034	0.813	0.047	0.805	0.034
JA	0.847	0.056	0.785	0.041	0.795	0.048	0.785	0.041
PA	0.738	0.120	0.545	0.097	0.661	0.080	0.545	0.097
DLS	0.867	0.034	0.821	0.014	0.826	0.020	0.821	0.014

### Prediction of Drug-Protein Interactions

In this section, new DPIs are predicted by the DLS method. First, all drug-protein interaction relationships are used to construct the DPI network. Then, The DLS method was used to predict new DPIs. According to the DLS method, assign a score value to each pair of drug-proteins that do not interact. All non-interacting drug-protein pairs are ranked from large to small according to the score value, and the probability of drug-protein pair interaction is judged based on the score value. The drug-protein interaction results predicted by the DLS method can be accessed at https://github.com/HNUBioinformatics/DLS.

**FIGURE 5:**
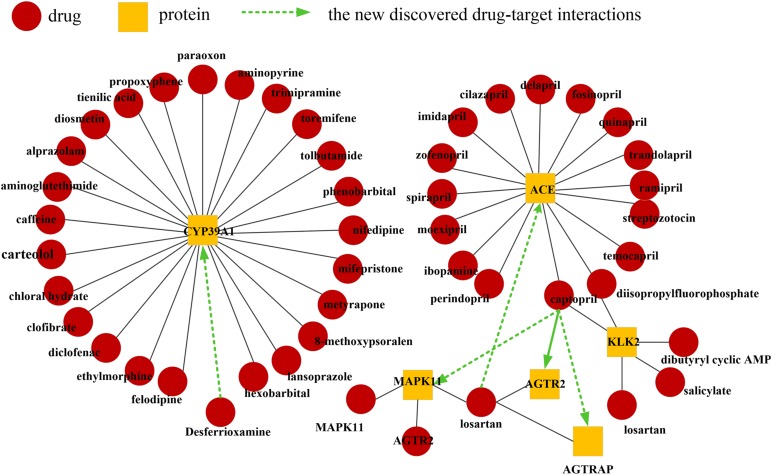
Network visualization of validated drug-protein interactions. The black lines indicate known drug-protein interactions. The green line between the drug (red circle) and protein (yellow square) indicates the novel discovered drug-protein interaction.

The predictions and the supporting evidences are shown in Table 2. As mentioned earlier, the drugs in this database are primarily responsible for cardiovascular disease. Here, we select drugs related to this type of system for analysis, including losartan and captopril. Recently, Maeda et al. (2013) found that AGTRAP could change the level of Insulin, while captopril could change the level of AGTRAP (Zhou et al., 2009, 2010; Cheng et al., 2012). A clinical study demonstrated that captopril can directly bind to angiotensin receptors (AGTR2) for hypotensive purposes (Takashi et al., 1997). The studies have shown that captopril may affect cardiomyocyte apoptosis and necrosis by acting on MAPK11 (Yang et al., 2018). Losartan may affect the microalbuminuria of diabetes by affecting the ACE gene, thereby affecting the production of angiotensin-converting enzyme (Tütüncü et al., 2001). The validated visualization of the drug-protein interaction network is shown in Figure 5. The evidences show that these cardiovascular-related drugs have successfully predicted new target proteins. Therefore, DLS method has great potential for predicting DPIs.

**TABLE 2:** The validated drug-protein interactions.

Drug	Protein	Rank	Evidence
Captopril	AGTRAP	4	Maeda et al., 2013; Ohki et al., 2018
Captopril	AGTR2	5	Yotsumoto et al., 1997
Desferrioxamine	CYP39A1	8	Ikeda et al., 2003
Captopril	MAPK11	127	Yang et al., 2018
Losartan	ACE	152	Tütüncü et al., 2001

## Discussion

### Comparison of the Methods

In the DPI network, the CN method and the JA method consider the overall network structure, and these methods do not analyze the interaction of drugs and proteins from the local structure. Drugs and proteins that do not directly interact may interact indirectly through many pathways. We define a pathway connected by two nodes as a secondary pathway. For example, *i* and *j* interact indirectly through the drug *i*-protein-drug-protein *j*, then *i* and *j* interact through the secondary pathway. DLS analyzes all secondary pathways of drugs and proteins and then obtains predicted values based on calculations for each secondary pathway. Compared with CN and JA, DLS starts from the local structure of the network and can investigate the interaction between drugs and proteins from a more detailed perspective. The PA method has the worst performance of all methods. The reason for its poor performance may be that PA simply studies the overall influence of nodes in the network. Our method achieves better performance than the three methods based on the local structure of the network and the degree distribution of each node.

### Method Composite

The above analysis found that both the CN method and the DLS method have high performance. We investigate whether the combination of CN and DLS can improve the performance of prediction. We integrate these two methods linearly, as shown in Equation 3. The CN method calculates the number of co-neighbors of drug-protein pairs, so the results of the CN calculations are all positive integers. The results calculated by the DLS method are all scores less than one. So we set a coefficient to correct the difference in weight between the two.

(3) $$S_{i\,j}^{\prime }=S_{i\,j}^{C\,N}+t*S_{i\,j}^{D\,L\,S}S_{i\,j}^{\prime }=S_{i\,j}^{C\,N}+t*S_{i\,j}^{D\,L\,S}$$

(4) $$S_{i\,j}^{\prime \prime }=S_{i\,j}^{C\,N}+h*S_{i\,j}^{J\,A}S_{i\,j}^{\prime \prime }=S_{i\,j}^{C\,N}+h*S_{i\,j}^{J\,A}$$

**FIGURE 6:**
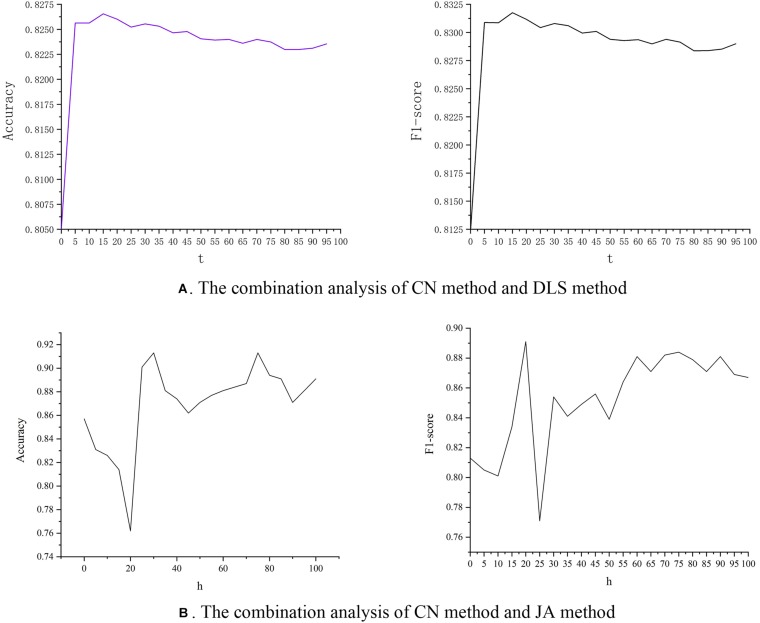
The relationship between parameters and predicted performance. **(A)** The combined performance of CN method and DLS method. **(B)** The combined performance of CN method and JA method.

We discuss the effect of parameter *t* on accuracy and F1-score. It can be seen in Figure 6A that as the value of *t* increases, the value of accuracy also increases. However, after *t* reaches 15, as the value of *t* increases, the value of accuracy starts to decrease. The value of F1-score also has the same trend. At the same time, we combined the CN method and JA method similarly, as shown in Equation 4. The experimental results are shown in Figure 6B. The results show that when parameter *h* is set 30, accuracy obtains the best performance, and when parameter *h* is set 20, F1-score get the best performance. This provides researchers with a new idea to improve prediction performance by combining the DLS method with other link prediction methods.

### Potential Application of Our Methods

At present, a large amount of biological data is represented in the form of networks, and how to mine information in the network has become a research hotspot. The network prediction method we developed can provide a fast and effective strategy for DPI prediction and drug repositioning to digest the large amounts of data. The weakness of this method is that DLS uses only the known information of the DPI network, there is no novel drug information for known proteins, so DLS could not predict the protein of novel drugs. Therefore, the link complement method should be developed to solve this problem and extend the method to other biological networks, such as gene-disease association networks, drug-disease prediction networks and protein-protein interaction networks.

## Conclusion

The prediction of DPIs is helpful for drug repositioning and the study of proteins in the pathogenesis. We propose a new DPI network link prediction method to predict unobserved DPIs, which is based on known drug-protein interaction networks and network local structures. Our method is different from existing DPI network prediction methods. It not only can make up for the shortcomings of structural similarity methods, but also does not require additional node information. The experimental results reveal that the DLS method is better than other comparison methods in predicting performance. In addition, the DLS method provides a novel idea for investigators to develop prediction accuracy by combining the DLS method with other link prediction methods. Case studies have shown that DLS can predict unobserved DPIs. However, DLS still has certain limitations. If there is no interaction between the drug and the protein, DLS cannot predict the new target protein of the drug, and how to solve this problem is our future work.

## Data Availability Statement

Publicly available datasets were analyzed in this study. This data can be found here: The MATADOR database (https://github.com/HNUBioinformatics/The-drug-protein-interactions-in-the-MATADOR-database).

## Author Contributions

WW studies drug-protein interaction networks and proposed a method using a network of local structure information to study drug-protein interactions. WW also provided the experimental methods. HL proposed a drug-protein interaction network and proposed a similarity approach to drug-protein interaction networks. YuaZ, DL, YW, and YuZ performed additional analysis of the results. All authors read and approved the final manuscript.

## Conflict of Interest

The authors declare that the research was conducted in the absence of any commercial or financial relationships that could be construed as a potential conflict of interest.

## Footnotes

1 http://matador.embl.de/
