# Supplementary material for: DLS: A Link Prediction Method Based on Network Local Structure for Predicting Drug-Protein Interactions
Source: Front Bioeng Biotechnol. 2020 Apr 24;8:330. doi: 10.3389/fbioe.2020.00330 (PMC7193019; doi:10.3389/fbioe.2020.00330)
Supplement: Supplementary file 1 [file Table_1.DOCX]

S-Table 1. The ratio of protein and drug by selected negative samples

| Index | The proportion of drugs | The proportion of proteins |
| --- | --- | --- |
| 1 | 0.859 | 0.835 |
| 2 | 0.851 | 0.847 |
| 3 | 0.874 | 0.845 |
| 4 | 0.861 | 0.834 |
| 5 | 0.865 | 0.845 |
| 6 | 0.874 | 0.856 |
| 7 | 0.860 | 0.847 |
| 8 | 0.863 | 0.826 |
| 9 | 0.859 | 0.847 |
| 10 | 0.868 | 0.831 |
| Average | 0.863 | 0.841 |


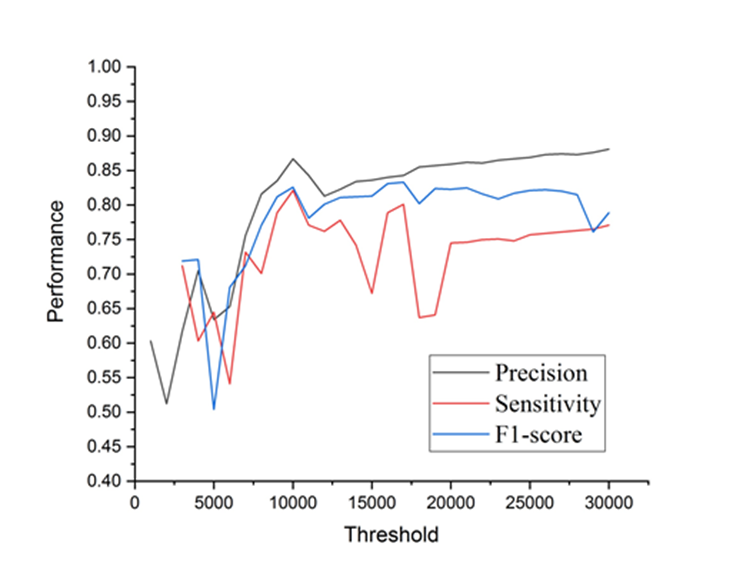


S-Figure 1. The relationship between performance and threshold. When the ranking threshold is set to 10,000, the performance is optimal.
